# Do Rapoport's Rule, Mid-Domain Effect or Environmental Factors Predict Latitudinal Range Size Patterns of Terrestrial Mammals in China?

**DOI:** 10.1371/journal.pone.0027975

**Published:** 2011-11-29

**Authors:** Zhenhua Luo, Songhua Tang, Chunwang Li, Jing Chen, Hongxia Fang, Zhigang Jiang

**Affiliations:** 1 Key Laboratory of Animal Ecology and Conservation Biology, Institute of Zoology, Chinese Academy of Sciences, Beijing, China; 2 Graduate School of Chinese Academy of Sciences, Beijing, China; Texas A&M University, United States of America

## Abstract

**Background:**

Explaining species range size pattern is a central issue in biogeography and macroecology. Although several hypotheses have been proposed, the causes and processes underlying range size patterns are still not clearly understood. In this study, we documented the latitudinal mean range size patterns of terrestrial mammals in China, and evaluated whether that pattern conformed to the predictions of the Rapoport's rule several analytical methods. We also assessed the influence of the mid-domain effect (MDE) and environmental factors on the documented range size gradient.

**Methodology/Principal Findings:**

Distributions of 515 terrestrial mammals and data on nine environmental variables were compiled. We calculated mean range size of the species in each 5° latitudinal band, and created a range size map on a 100 km×100 km quadrat system. We evaluated Rapoport's rule according to Steven's, mid-point, Pagel's and cross-species methods. The effect of the MDE was tested based on a Monte Carlo simulation and linear regression. We used stepwise generalized linear models and correlation analyses to detect the impacts of mean climate condition, climate variability, ambient energy and topography on range size. The results of the Steven's, Pagel's and cross-species methods supported Rapoport's rule, whereas the mid-point method resulted in a hump-shaped pattern. Our range size map showed that larger mean latitudinal extents emerged in the mid-latitudes. We found that the MDE explained 80.2% of the range size variation, whereas, environmental factors accounted for <30% of that variation.

**Conclusions/Significance:**

Latitudinal range size pattern of terrestrial mammals in China supported Rapoport's rule, though the extent of that support was strongly influenced by methodology. The critical factor underlying the observed gradient was the MDE, and the effects of climate, energy and topography were limited. The mean climate condition hypothesis, climate variability hypothesis, ambient energy hypotheses and topographical heterogeneity hypotheses were not supported.

## Introduction

Spatial patterns of species range sizes and their underlying mechanisms at large scales are hot topics in macroecology, biogeography and biodiversity conservation [1]–[3]. Rapoport's rule, once considered to be the second robust biodiversity rule, predicts that species occupy broader ranges at higher latitudes, and as such, mean latitudinal range size enlarges with latitude increase [4]. Several studies have investigated range size gradients among mammals in the New World [5]–[7], Palearctic [8], Africa [9], [10], Australia [11], [12], or at the global scale [13], in order to test Rapoport's rule and uncover the factors shaping these patterns. Despite its applicability to plants [4], [14], invertebrates [15], fish [16], birds [17], mammals [7], [8], and both amphibians and reptiles [18], the validity of Rapoport's rule has been the subject of considerable scrutiny and debate [16], [19], [20]. Some studies have failed to found support for this rule, or detected results consistent with the rule only over fairly narrow latitudinal limits [20], [21]. Thus, it is necessary to carry out more detailed research to clarify the role that biogeographical factors have on range size pattern, and to clarify just how robust such findings are in light of variation in methodology, the mid-domain effect (MDE), and environmental heterogeneity [21]–[23].

The methods used to depict latitudinal gradients in range size greatly influence differences in the magnitude and perceptions of the measured patterns [18], [22], [24]. Thus, it is valuable to compare the predictions of several methods in detail [18], [25], [26]. Steven's method [4], the mid-point method [27], Pagel's method [28] and the cross-species method [8] have been employed frequently in recent decades to evaluate Rapoport's rule, and often provide information that complements different perceptions of the patterns [18], [26]. The MDE, as a null model, offers a simple non-biological explanation of the limit of geometric constraints on species geographical ranges without influences accounting for the environmental variation [29]–[31]. The MDE predicts a massive overlapping at the centre within a domain, and larger mean range sizes near the centre than in peripheral areas [3], [15], [30], [32]. Mean environmental conditions and variability of climate are critical in setting species' breadth of tolerance, and both higher climate condition and enhanced climatic stability promote reduced distribution sizes [4], [19], [33]. The mean climate condition hypothesis and climate variability hypothesis predict monotonic increases of mean range size with latitude or a hump-shaped pattern [4]. Energy and topography affect distribution, population size, migration, and/or specialization of individual species, and therefore contribute to overall change in range size pattern [34]–[36]. The ambient energy hypothesis and topographical heterogeneity hypothesis predict larger ranges under higher energy regimes and complex topography [34]–[36].

Ideally, studies on species distribution pattern should encompass large areas at macro-scale; misleading results may be obtained if research is limited to an overall area that provides only partial coverage [37]. China is one of the top twelve mega-biodiversity countries in the world, with a vast land area, wide latitude range, complex terrain, diverse climate, and extensive field surveys on mammalian distribution available over recent decades [38]. These data provide an excellent opportunity to study the latitudinal range size pattern of mammals and the impacts of ecogeographic factors on the resultant pattern. Moreover, as such research is lacking in China, our study is urgently needed given the critical nature of understanding range size patterns as a prelude to effectively conserving biodiversity. We aimed to: (1) reveal the latitudinal mean range size pattern of terrestrial mammals and create a range size map of terrestrial mammals in China; (2) test the Rapoport's rule using Steven's, mid-point, Pagel's and cross-species methods; (3) evaluate the effects of methodology, the MDE and environmental factors on mammalian range size pattern.

## Methods

Our study covered the mainland and two largest islands (Taiwan and Hainan islands) of China. Thus limits for inclusion of data in the present study spanned 18 through 54° N latitude and 73 through 135° E longitude.

### Species ranges

An exhaustive database of distributions of 625 mammal species, encompassing 13 orders, 55 families and 235 genera, was originally complied following IUCN *et al*. (2004), Sheng *et al*. (2005), Pan *et al*. (2007) and the Vertebrate Species Information Database of our research group [39]–[42]. We excluded primarily marine and aquatic species, whose geographical ranges are unique from terrestrial mammals. We digitized the range maps and updated them according to comprehensive literature, faunistic atlases, nature reserve biodiversity survey reports, documents of museum collections and field survey records from our laboratory. Numerous zoologists were also consulted to modify the database. One-hundred and ten species that were subject either to taxonomic disputes or lacking comprehensive distributional information were removed from the overall data set, leaving 515 terrestrial mammal species in our analyses.

For each species, we recorded the maximum and minimum latitudes of its distribution. Further, the mid-point and latitudinal range of each species was calculated as the average and difference between the maximum and minimum latitudes respectively. To evaluate the relationship between mean species range and latitude, the total latitudinal gradient was divided into eight bands of 5° latitudinal intervals. In addition, we rasterized the range maps into equal-area grids of 100 km×100 km [37].

### Environmental predictors

To evaluate the effects of environmental factors, we used nine predictive variables that were collapsed into the four grouped environmental variables reported below, all of which were processed into 100 km×100 km equal-area grids. Coastal cells were excluded if they contained <50% of the land masses.

#### (1) Mean climate condition

The data on annual mean temperature (AMT, °C) and annual precipitation (AP, mm) were compiled at a 1 km×1 km resolution from WorldClim 1.4 at http://www.worldclim.org/ [43].

#### (2) Climate variability

We used temperature annual range (TAR, °C), temperature seasonality (TS, °C) and precipitation seasonality (PS, mm) as predictors of climate variability. These data were compiled at a 1 km×1 km resolution from WorldClim 1.4 at http://www.worldclim.org/ [43].

#### (3) Ambient energy

Potential evapotranspiration (PET, mm) for the years 1950–2000 were overlain on 1 km×1 km grids using data from CGIAR Consortium for Spatial Information (CGIAR-CSI) at http://www.csi.cgiar.org/ [44]. We also included the annual mean normalized difference vegetation index (NDVI) as a predictor of ambient energy for 1950–2000, using 1 km×1 km resolution data from http://www.data.ac.cn/ [45]. We calculated the annual mean NDVI by averaging these data.

#### (4) Topography

We extracted altitude (ALT, m) and altitude range (ALR, m) data from a global digital elevation model (CGIAR-CSI at http://srtm.csi.cgiar.org/) with 1 km×1 km resolution as indicators of topography and its heterogeneity [46], [47].

### Analyses

We examined the relationship between mean latitudinal range size and latitudes among 5° bins using Steven's method [4], the mid-point method [27], Pagel's method [28] and the cross-species method [8]. Linear and 2nd order polynomial fits were calculated, and the fit with the highest *R* ^2^ was selected to represent the relationship. Rapoport's rule is supported where the relationship between those variables is positive [15]. Moreover, we assigned each species range raster with its latitudinal range, and calculated the arithmetic mean in each 100 km×100 km grid cell [20], to reveal any spatial pattern in range size and ultimately, analyze the relationship between range size and environmental factors.

We tested the impacts of two factors: (1) the MDE and (2) environmental effects. First, we detected the MDE by comparing the observed latitudinal range pattern with the null model built by reshuffling species ranges based on an empirical distribution range model [30], [31] parsed in 5° latitudinal bands system. The simulation was performed using a Monte Carlo algorithm and implemented in the modules Mid-Domain Null [48] and Range Model 5 [49]. We ran 10000 Monte Carlo simulations of empirical range sizes sampled without replacement to ensure that all species were reshuffled [15]. The mean latitudinal range size from those 10000 simulations was considered to be the prediction of the null model [50], and a linear regression of the empirical mean range sizes and the null model was carried out to interpret the impact of the MDE [51], [52]. For the linear regressions, we checked normality (K–S test) and homoscedasticity (Levene's test) of the data, all of which detected no significant departure from either normality or homoscedasticity (all *P* >0.05). Second, we processed generalized linear models (GLMs) between mean latitudinal range sizes and four groups of environmental variables separately over the 100 km×100 km quadrat system to explain the environmental impacts on range size gradient [15]. To ameliorate the problems of high correlations between explanatory variables (Pearson's correlation coefficient >0.7), we used stepwise procedures in the GLMs. The relationships among variables, latitudes and mean latitudinal range sizes were also determined using Pearson's correlation coefficients [15].

Our statistical analyses were carried out in SAS Version 9.1 and SPSS Version 13.0. The spatial analyses were conducted in ESRI ArcGIS 9.2.

## Results

### Species latitudinal range size distributions

**Figure 1:**
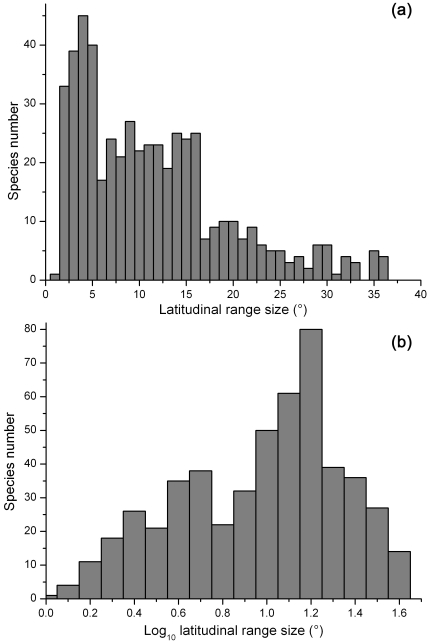
Species latitudinal range size distribution for terrestrial mammals in China . (a) untransformed latitudinal range size; (b) log_10_-transformed latitudinal range size.

The mean latitudinal range size of terrestrial mammals in China was 11.01±8.13° (mean±SD; applies to all subsequent values) (*n* = 515), with the median of 9.58°. The distribution of range size was formally right-skewed (Fig. 1(a)), and the log_10_ transformed range sizes were not normal but assumed a modestly left-skewed distributed (Fig. 1(b)). Only 17 (3.3%, the percentage of the total species number; applies to all subsequent values) and 34 (6.6%) species had ranges of >30° and <2°, respectively (Fig. 1(a)). More than three quarters of the species (79.8%, 411 species) occupied ranges of 2°–20°, and 90.1% species (464 species) occupied 2°–30° of latitudes (Fig. 1(a)).

### Spatial pattern of latitudinal range size

**Figure 2:**
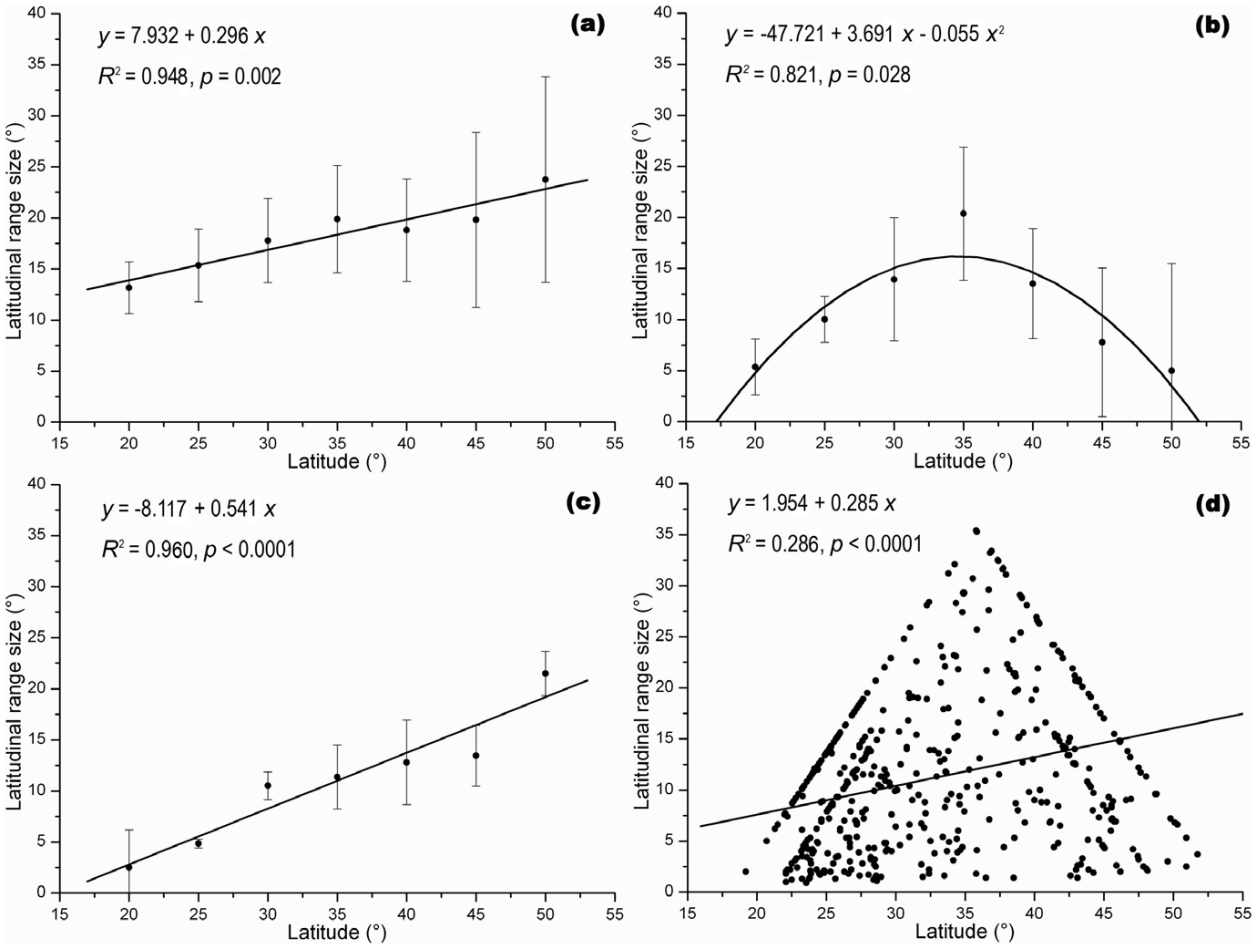
Mean latitudinal range size of terrestrial mammals among latitudes in China. Solid lines represent the fitted correlations between mean latitudinal range sizes and latitudes: (a) Steven's method (sample size within each 5° band (left to right): 206, 233, 201, 161, 161, 124, 56); (b) mid-point method (sample size within each 5° band (left to right): 114, 138, 83, 64, 74, 37, 5); (c) Pagel's method (sample size within each 5° band (left to right): 37, 119, 94, 65, 45, 86, 69); (d) cross-species method (total sample size was 515).

The latitudinal range size patterns predicted by Steven's, Pagel's and the cross-species methods were related positively to latitudes, which conforms to Rapoport's rule (Fig. 2). Steven's method showed that the range size was between 13° to nearly 25°, and attained its minimum between 15° N and 25° N, increasing northward (β = 0.296, *p* = 0.002, *R* ^2^ = 0.948; Fig. 2(a)). Pagel's method revealed a similar pattern, but with a steeper slope than Steven's method (β = 0.541, *p* <0.0001, *R* ^2^ = 0.960; Fig. 2(c)). Application of Pagel's method resulted in a mean range size of <3° in the southernmost band (Fig. 2(c)). The scatter diagram representing results obtained via the application of the cross-species method revealed a mean range size between Steven's and Pagel's methods, with a positive latitudinal gradient explained by limited variation in range size pattern (β = 0.285, *p* <0.0001, *R* ^2^ = 0.286; Fig. 2(d)). Application of the mid-point method, however, revealed a hump-shaped relationship, peaking in the vicinity of 30° N−35° N with significant declines both to the north and south (*p* = 0.028, *R* ^2^ = 0.821; Fig. 2(b)). These latter findings do not support Rapoport's rule, and suggested that the maximum and minimum of mean range size were 20° and 5° respectively (Fig. 2(b)).

**Figure 3:**
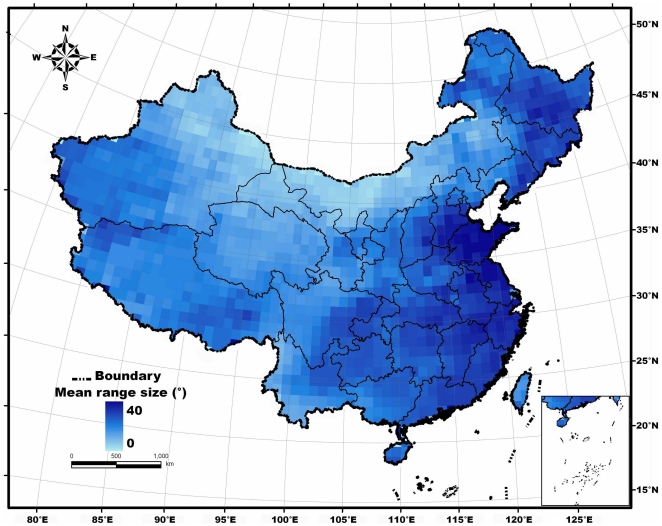
Geographical pattern of mean latitudinal range size of terrestrial mammals in China, resolved to 100 km×100 km. The color gradient represents the mean latitudinal range extent in each grid cell.

The latitudinal range size map revealed no pattern consistent with Rapoport's rule (Fig. 3). It showed larger mean range sizes between 25°N and 40°N. The eastern and southeastern coastal areas were characterized by the biggest species ranges in China, followed by the northeastern, central and southern part of the country. By contrast, the smallest ranges were found along the northern border. The mean range size in the vast western inland remained relatively small, and indicated a slight increase toward the west (Fig. 3).

### Impact of the MDE on mean range size pattern

**Figure 4:**
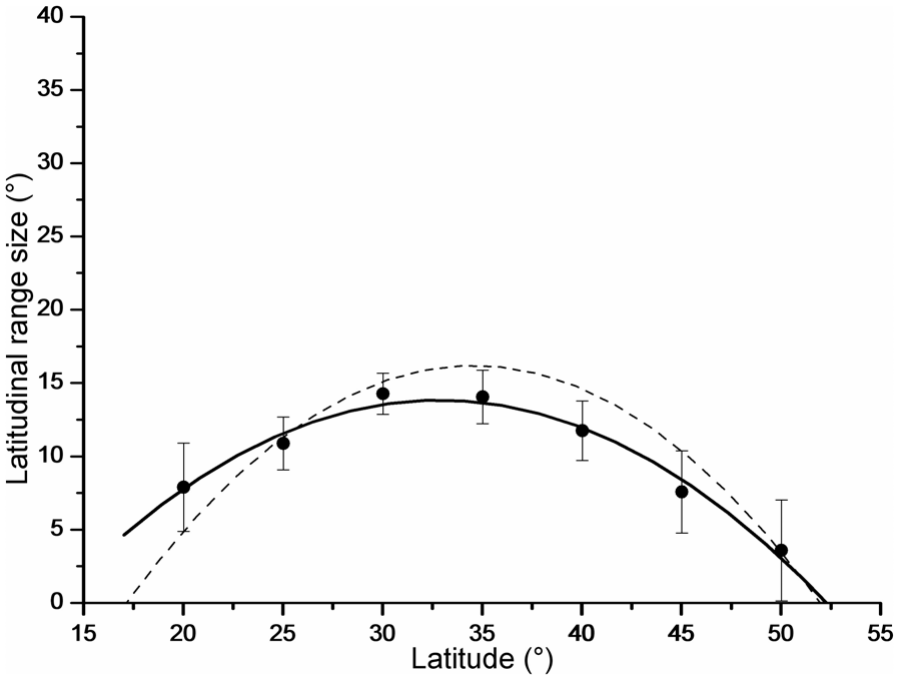
Simulated mean latitudinal range size in each 5° band from 10000 Monte Carlo simulation runs (black points, using mid-point method). The black (*y* = −24.566+2.409*x*–0.037*x* ^2^, *R* ^2^ = 0.971, *p* <0.0001) and dotted lines show the 2nd order polynomial fits of the predicted and empirical mean latitudinal range sizes respectively.

Our results indicated an obvious and important impact of MDE in shaping the latitudinal gradient of range size. The Monte Carlo simulations (null model) indicated a hump-shaped range distribution of size variation (*R* ^2^ = 0.971, *p* <0.0001; Fig. 4). The highest mean range size (nearly 15°) emerged among the mid-latitudes (25° N–35° N), whereas the smallest range was located near the northern (smaller than 5°) and southern (nearly 8°) borders of China (Fig. 4). The MDE revealed a similar gradient to the mid-point method, but with a gentler slope (Fig. 4). The linear regression (β = 0.623, *p* = 0.005, *R* ^2^ = 0.802) revealed that the null model explained 80.2% of the range size variation.

### Impacts of environmental factors on mean range size pattern

The relationships of environmental variables and latitude indicated that mean climate condition, ambient energy and topography decreased with increasing latitude, but climate variability was positively correlated with latitude (Table 1). AMT (*r_l_* = −0.831) and AP (*r_l_* = −0.666) were strongly negatively related to latitude, whereas, TAR (*r_l_* = 0.964) and TS (*r_l_* = 0.957) were strongly positively related to latitude (Table 1). The residual variables had correlation coefficients of <0.6 (Table 1). Mean climate conditions and ambient energy were positively correlated with range size variation, while climate variability and topography were negatively correlated with range size variation (Table 1). The relationships between AP (*r_m_* = 0.522) and NDVI (*r_m_* = 0.453) with range size were strongly positive, whereas, the residual coefficients of those relationships were <0.4 (Table 1).

**Table 1:** Pearson's correlations of environmental factors with mean range size (***r_m_***) and latitude (***r_l_***).

Predictive variables	*r_m_*	*r_l_*
Mean climate condition		
AMT	0.357	−0.831
AP	0.522	−0.666
**Climate variability**		
TAR	−0.307	0.964
TS	−0.188	0.957
PS	−0.236	0.351
**Ambient energy**		
NDVI	0.453	−0.082
PET	0.177	−0.366
**Topography**		
ALT	−0.302	−0.165
ALR	−0.143	−0.342

All the correlations were statistically significant (*P* <0.0001).

The environmental factors were not deterministic of range size, but contributed moderately to the observed range size gradient, in that the GLMs explained only limited variation (*R* ^2^ <0.30, Table 2). The explanatory power of the mean climate conditions to range size was 27.7%, and AP accounted for 27.1% of the variation in range size. Climate variability and ambient energy explained 28.7% and 21.7% of that variation, while, TS and NDVI explained 18.6% and 20.5% respectively. Topography had the lowest contribution, with ALT accounting for only 9.2% of the variation in range size (Table 2).

**Table 2:** Stepwise generalized linear models (GLMs) between the four groups of environmental variables and mean species range sizes.

	β	*t*	*p*	Adjust *R* ^2^
**Mean climate condition: ** ***F*** **_2, 909_ = 268.065, ** ***R*** **^2^ = 0.277, ** ***P*** ** <0.0001**				
AP	0.634	17.194	<0.0001	0.271
AMT	−0.144	−3.908	<0.0001	0.277
**Climate variability: ** ***F*** **_3, 908_ = 184.985, ** ***R*** **^2^ = 0.287, ** ***P*** ** <0.0001**				
TAR	−2.158	−20.342	<0.0001	0.091
TS	1.876	18.443	<0.0001	0.277
PS	0.092	3.333	0.001	0.287
**Ambient energy: ** ***F*** **_2, 909_ = 193.284, ** ***R*** **^2^ = 0.217, ** ***P*** ** <0.0001**				
NDVI	0.437	18.224	<0.0001	0.205
PET	0.113	4.732	<0.0001	0.217
**Topography: ** ***F*** **_1, 910_ = 142.454, ** ***R*** **^2^ = 0.092, ** ***P*** ** <0.0001**				
ALT	0.305	−11.935	<0.0001	0.092

β, coefficient of generalized linear model of each variable.

## Discussion

### Species latitudinal range size distributions

The distribution of latitudinal range size of terrestrial mammals in China was right skewed, with small ranges for the majority of species (Fig. 1(a)). Most species occupied medium sizes of ranges, and very few species enjoyed very broad or very limited ranges. This result is consistent with those identified among most animal assemblages [17], [53], [54]. The departure from a normal distribution (left skewed pattern, Fig. 1(b)) of logarithmic ranges also parallels that documented in previous studies of birds and mammals [17], [53]–[55]. Such a pattern may be caused by either an absence or excess of rare species in the database [23], [56], [57], along with range size limitations imposed by the limited dispersal abilities of species, precluding their migration to all major land masses >[17].

### The effect of methodology on testing Rapoport's rule

The method used to test Rapoport's rule had a pronounced impact on the results obtained [18], [19], [58], [59]. The averaging of range sizes, in particular, moderates the difference in the magnitude of range size pattern [18]. Most studies have used one-dimensional statistical approaches based on scatter plots and correlation analyses between range sizes and latitudes across individual species, or directly map the mean range size over continents [17], [18], [20], [23].

In this study, the results obtained with Steven's method, Pagel's method and the cross-species method revealed significant overall positive trends, which support Rapoport's rule (Fig. 2). The mid-point method, however, suggested that the range size-latitude relationship was non-linear with a peak in range size at intermediate latitudes (Fig. 2). Similar gradients were reported in previous research on vascular plants of Taiwan and Mt. Shennongjia, China, which, like our study, contrasted the results of the same four methods [58], [59]. Gaston *et al*. (1998), Bhattarai & Vetaas (2006), Feng *et al*. (2006) and Hausdorf (2006) all reported that different methods could lead to variation in the results obtained [14], [19], [60], [61]. Such findings not only reveal complementary information on species range patterns, but reflect the sensitivity of such techniques to the different methods of reducing the original information to basic data for analyses [18], [25]. Steven's method is susceptible to problems of autocorrelation [27]. The mid-point and Pagel's methods are strongly influenced by the geometrical boundary, and can produce abnormal results when limited data are available in some latitudinal bins [8], [62]. The cross-species method is sensitive to the underlying species richness pattern [8], [58]. Where statistics are being used as an indicator of the relationship between latitude and species range size, it is important to take the impact of the methodology underlying that generation of that statistic into account [18].

The mid-point method provided results that corresponded most directly with those apparent from visual examination of the mean range size map, but did not support Rapoport's rule (Fig. 3). The map showed larger ranges in the mid-latitudes (25°N–40°N), especially in the eastern and southeastern parts of China. Range sizes in the tropics (sub-tropics) and boreal zones (north to 40°N) were much smaller.

### Impact of the MDE on mean range size pattern

Colwell *et al*. (2004) argued that the mid-domain peaked pattern may occur in the absence of any contributing heterogeneity in underlying environmental factors [31] particularly where geometric constraints themselves act as barriers against species dispersal [63]. It has been suggested, however, that the MDE alone may not adequately account for this pattern, and thus, the integration of non-random factors would be required to explain variation in range size with changing latitude [3]. Geographic boundary effects and environmental, topographical and biological variations typically interact strongly, and the apparent explanatory power of the MDE may be an indirect product of the effects of climate, ambient energy or geographical complexity on species' distributions [3]. Considering the deviation of the empirical range size gradient from the null model proves valuable in disentangling the impacts of these factors [30]. Furthermore, as the MDE could change species' immigration/emigration mode in an area, it could also modify the range size pattern through the “Rapoport-rescue effect” [32].

The results of our study revealed a hump-shaped latitudinal range size gradient, and detected marked impacts of the MED (Fig. 4). Our results also supported the expectations of the null model, implying significant contributions of geometric constraints or geographic boundary effects. Our findings are in accord with those from previous research on flowering plants [64], the New World mammals [48], [65], [66], birds [67], [68], African vertebrates and insects [69] and marine species [70], [71]. By contrast, Bokma *et al*. (2001), Diniz-Filho *et al*. (2002), Hawkins & Diniz-Filho (2002), Sanders (2002) and Moreno *et al.* (2008) reported findings that were inconsistent with the MDE [15], [32], [72]–[74].

### Impacts of environmental factors

Mean range size may be correlated with environmental variables. Climate and the variation therein have been reported to be deterministic of species range sizes [4], [21], [75], in that environmental variation sets the minimum tolerance range for a species, and the interaction between mean climatic conditions and variation generate the commonly observed pattern of increasing range size with increasing latitude [19], [21]. If mean climate condition and climate variability hypotheses operated, species occupying areas at higher latitudes would be subject to selection expanding their tolerances and range sizes, so as to allow survival in the face of greater environmental variation and at lower mean climatic condition [19], [33]. The results of research on both the continental and global scale involving plants, fish, birds and mammals support that contention [4], [8], [28], [54]. Ambient energy determines the baseline environmental capacity for species diversity, and greater heterogeneity in spatial and topographical habitat structure could permit finer subdivision of limiting resources and, hence, promote greater specialization of species [1], [76]. Thus, the ambient energy hypothesis could account for the co-existence of a greater number of species in equatorial regions, with presumably more frequent interaction among species, and thereby result in increasing range size with increasing latitude [77]–[79]. Topographical heterogeneity hypothesis predicts a negative relationship between topographical variation and range size. Studies on plants, invertebrates, fish, reptiles and birds provide data that conform to these species richness and distribution patterns [80]–[83].

In this study, climate, ambient energy and topography contributed minimally to the observed variation in range size, while AP, TS and NDVI accounted for substantial proportions of the observed variation (Table 1, Table 2). Our results did not support the mean climate condition, climate variability, ambient energy or the topographical heterogeneity hypotheses, in that all of these mechanistic hypotheses predict narrower range sizes at higher latitudes (Table 1, Table 2). That said, our findings may well be accounted for, at least in part, by the “Rapoport-rescue effect” [4], [55], [84]. If species at different latitudes have similar underlying dispersal abilities, species at lower latitudes may disperse outside what could be considered optimum habitat than species residing in areas at higher latitudes [4], [84]. This difference in range size expansion, which occurred as a result of dispersal, might account for latitudinal range size gradient we detected.
